# XIAP at the crossroads of cell death and inflammation

**DOI:** 10.18632/oncotarget.25363

**Published:** 2018-06-08

**Authors:** Domagoj Vucic

**Affiliations:** Genentech, South San Francisco, CA, USA

**Keywords:** IAP, XIAP, antagonist, RIP2, NOD2, Autophagy

Accurate regulation of cell death is crucial for proper development and homeostasis of organisms. Consequently, deregulated cell death can lead to developmental defects, neuropathies, infections, inflammatory diseases and cancer. Many cell death regulators have additional functions, including important roles in cell division, metabolism, inflammatory signaling pathways and tumor biology. Inhibitor of apoptosis (IAP) proteins were initially identified as inhibitors of cell death during viral infection, but are no exception to that paradigm [1, 2]. Several IAP proteins, including XIAP (X chromosome-linked IAP), are ubiquitin E3 ligases with seminal roles in diverse signaling pathways [3]. The most prominent IAP ubiquitination substrates, RIP1 and RIP2 proteins, are modified by c-IAPs (cellular IAPs) and XIAP E3 activity in TNF and NOD2 signaling, respectively [3]. XIAP also influences RIP1 ubiquitination and RIP3 dependent cell death and IL-1β secretion in response to TNF [4].

**Figure 1:**
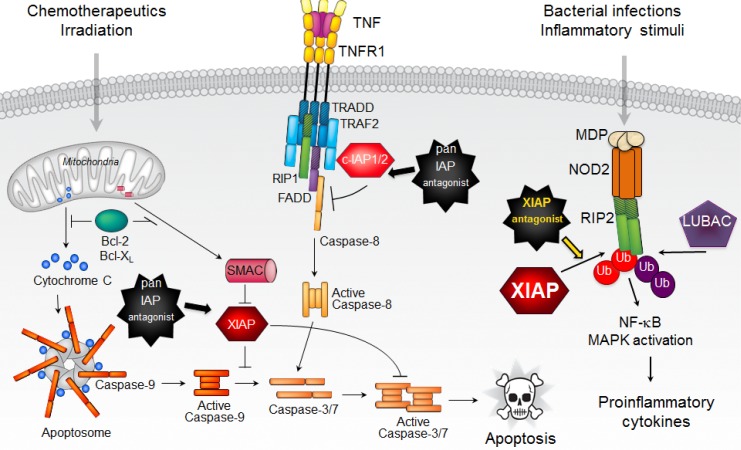
XIAP regulates apoptotic and inflammatory signaling pathways XIAP is an inhibitor of caspases 3, 7, and 9, which allows it to block both mitochondrial and death receptor mediated cell killing. By serving as an E3 ligase for RIP2, XIAP is a critical mediator of NOD2/RIP2 inflammatory signaling. Consequently, XIAP selective antagonists can efficiently inhibit NOD2 pathway.

XIAP is the only endogenous mammalian inhibitor of caspases, which function as executioner proteases in cell death pathways [2]. It uses its BIR2 domain and the linker region between the BIR1 and BIR2 domains to bind the substrate-binding groove of active caspases 3 and 7, and its BIR3 domain to bind the conserved four amino-terminal amino acids of the p12 small subunit of processed caspase-9 (Figure 1). Thus, not surprisingly, initial efforts at targeting IAP proteins in cancer were focused on antagonizing XIAP binding to and inhibition of caspases. However, although XIAP was the main target, most IAP antagonists had pan-IAP activity with no significant selectivity for XIAP [2]. Discovery and characterization of c-IAP1/2 selective and, more recently, XIAP selective antagonists confirmed that XIAP selective antagonism is not sufficient to promote cell death in cancer cells [2, 5]. Interestingly, the main effect of pan-IAP antagonists was the activation of c-IAP1/2 E3 activity leading to their auto-ubiquitination, proteasomal degradation and subsequent activation of canonical and noncanonical NF-kB signaling and production of inflammatory cytokines [2]. The most important cytokine produced was TNF, which stimulated cell death in the absence of degraded c-IAP1/2. This unique feature of IAP antagonists enabled their single agent activity in some cancer cell lines and tumor models [2]. Nevertheless, overall, this activity was not robust enough and, coupled with a lack of clear predictive biomarkers, IAP antagonists have not reached their potential as a cancer treatment option through the activation of cell death.

In the case of XIAP, genetics pointed researchers in the right direction because XIAP knockout mice lack cell death defects but fail to mount an immune response to Listeria infection or treatment with peptidoglycans such as muramyl dipeptide (MDP), which is made by gram positive and negative bacteria [1]. In addition, mutations in *XIAP* are associated with inflammatory diseases such as XLP-2 and VEO-IBD [1, 6]. Recognition of MDP leads to NOD2 activation and recruitment of RIP2 and XIAP, which promotes RIP2 K63-linked ubiquitination. These initial events allow the recruitment of LUBAC, linear ubiquitination of RIP2 and activation of NF-kB and MAPK signaling resulting in the production of inflammatory cytokines and chemokines [7]. Although several other E3 ligases bind to and can promote RIP2 ubiquitination, XIAP is instrumental for efficient NOD2- RIP2 mediated signaling [5, 7, 8]. Interestingly, although c-IAP1/2 are only tangentially relevant for NOD2 mediated signaling and RIP2 ubiquitination, they do contribute to NOD2-dependent autocrine TNF signaling and amplification of cytokine production in vivo [8]. Absence of XIAP, on the other hand, cripples NOD2 signaling [7]. Similarly, XIAP selective antagonists also severely blunt NOD2 mediated activation of NF-kB and MAPK signaling and cytokine/chemokine production [5]. They achieve that task by disrupting the direct physical interaction between the E3 ligase XIAP and its substrate, kinase RIP2 (Figure 1). Disruption of XIAP-RIP2 binding prevents RIP2 ubiquitination and the assembly of the NOD2 signaling complex, thus precluding production of inflammatory cytokines.

XIAP selective antagonists are well positioned for intervention in NOD2-mediated pathologies because, unlike pan IAP antagonists, they do not activate cell death, c-IAP1/2 autoubiquitination and proteasomal degradation, or NF-kB signaling [5]. XIAP selectivity is possible because of the uniqueness of the XIAP BIR2 domain that binds to RIP2. Past drug discovery efforts on pan IAP antagonists have produced reagents with favorable potency and pharmacological properties allowing clinical trials in cancer patients [2]. The hope is that future optimization of XIAP selective antagonists will also yield promising agents for therapeutic intervention in NOD2- mediated diseases such as Crohn’s disease, sarcoidosis and Blau syndrome.
